# The Clinical Manifestations and Treatment of Coliuria in Children

**Published:** 1914-12

**Authors:** R. G. Gordon


### THE CLINICAL MANIFESTATIONS AND TREATMENT OF COLIURIA IN CHILDREN.

BY

R. G. Gordon, M.D., B.Sc., M.R.C.P. Ed.

In adults coliuria is an obvious and common complaint, which is chiefly interesting by reason of the difficulty in establishing a lasting cure, but I do not suppose that there is any affection of childhood in which one is so frequently led astray.

In adults one may say that one's attention is almost always drawn to the urinary tract, and that in consequence one obtains a sample of the urine and at once the diagnosis is plain ; but in children there may be, and quite often are, no urinary symptoms, and although routine examination of the urine ought theoretically to be carried out, everyone knows how extremely difficult it is to obtain an uncontaminated sample from the infant and young child, especially in the female sex. For these reasons the diagnosis is often very difficult, and the condition is missed over and over again. When one comes to examine the clinical manifestations, one is at once struck by the fact that they present a confused heterogenous group of symptoms, some of which are exhibited by one case and some by another, and I propose to try to arrange these symptoms into groups, so that one may classify one's cases, giving illustrative cases of the various types.

Firstly, cases may be divided into acute and chronic, of which the acute are the most numerous, present the most interesting pictures, and are most amenable to treatment.

From the observation of a series of cases in a children's hospital, and from a study of the literature, five groups of acute cases suggest themselves, viz. :—

1. Those presenting general symptoms without reference to any special symptom.
2. Those with nervous symptoms predominating.
3. Those with pulmonary symptoms predominating.
4. Those with gastro-intestinal symptoms predominating.
5. Those with urinary symptoms predominating.

For example—

A girl aged five years and four months was sent to hospital for diagnosis. Six weeks before admission she had slight pain and inflammation of the throat, adenoids having recently been removed. The temperature went up to 105°, and had continued swinging ever since. Three days after the onset there was severe pain in the back lasting twelve hours. The bowels had been confined, the appetite poor, and the child irritable and difficult to manage.

On admission the child looked pale and ill, but physical examination yielded nothing, and there were no urinary symptoms, but on examining the urine it was found to be acid, to contain epithelial and pus cells, and on cultivation yielded a pure growth of *Bacillus Coli*.

This case I should place in the first group, and such cases are marked by a sudden onset of high fever, often accompanied by rigors, which, by the way, are very suggestive of coliuria when they occur in children, by convulsions or sudden collapse. If untreated, the temperature may swing for weeks, and the charts often simulate typhoid or malaria. There is often marked restlessness and irritability and sometimes pronounced muscular tenderness, but no symptom pointing to any special system.

The second group specially tends to simulate meningitis, and these cases may be delirious, convulsed or drowsy, and even comatose. They may show neck rigidity, strabismus, and even Kernig's sign, while extreme irritability and frequent screaming fits seem to complete the picture of meningitis. There are no urinary symptoms, but the urine is found to contain pus and *Bacillus Coli*, and on treatment they recover.

The following is a good example :—

A girl aged seven and a half months, rather constipated but otherwise healthy, was suddenly taken ill one evening with a temperature of 104°. Next morning she was unconscious, with the body extended and rigid. This latter condition lasted an hour, and she was semi-conscious till afternoon, when she had right-sided convulsions with deviation of the eyes to the right, lasting for three hours. She was given bromide and chloral per rectum, and then chloroform was administered for one hour, after which she slept for six hours. On waking the temperature was 100°, and she was paralysed in the right arm and leg, but not in the face. After a few days the paralysis passed off. Four days later the temperature rose to 104° without apparent cause, and continued swinging for three weeks, with no rigors or fits, but with periods of unconsciousness at intervals, in one of which the pulse rate increased to over 200. All this time there were no urinary symptoms, but one day the nurse noticed peculiar staining on the napkins, and the urine was collected and examined. This was found to contain pus and *Bacillus Coli*, so she was treated accordingly, and made an uninterrupted recovery. Five years after she is a healthy, intelligent child, and has had no further cerebral trouble whatever.

The third group leads one to expect that some pulmonary trouble, pneumonia or acute bronchitis, is about to develop. The onset is attended with tachypnœa and sometimes dyspnœa, together with high temperature and malaise. A few râles may be heard at the bases, but the signs never develop though the symptoms persist, and it is not until the urine is examined that the true cause is elicited.

Thus :—

A girl aged eleven months was admitted to hospital with a history of cough, dyspnœa, and twitching of the eyes of two days' duration, the onset being accompanied by vomiting. On examination the child presented a typical pneumonic appearance, with inverted breathing, but beyond a few râles there were no signs in the lungs and no consolidation was ever present. The urine, however, showed pus and bacilli, and treatment directed to this had a satisfactory result.

In the fourth group the onset is attended by severe colicky pains in the abdomen, vomiting, obstinate constipation, or in some cases diarrhœa. The appetite is lost, and in a few cases jaundice is present. There are, however, no localising signs till the urine is examined.

Thus :—

A girl aged eleven months was admitted to hospital with diarrhœa and vomiting, accompanied by screaming fits, attributed to colic. She was irritable and feverish, and her condition had resisted the usual methods of treatment, but on the urine being examined a condition of coliuria was discovered, and recovery rapidly ensued on the treatment of this condition.

Fifthly, we have the cases which do present urinary symptoms, which of course form the largest individual group, but from such statistics as are available they would only seem to amount to 50 per cent. of the whole. In such cases there may be a palpable tender kidney which may vary in size as in hydronephrosis, and they all present symptoms of cystitis—frequency and screaming during the passage of urine. Such symptoms may be intermittent, but continue unless treatment is adopted. It is hardly necessary to cite such a case, as they are quite common and the diagnosis is fairly obvious.

So much for acute cases, and in passing it may be remarked that all these symptoms must be attributed to the circulation of toxins produced by the bacilli, and evidence of this is afforded by the results of treatment. It is unnecessary to dwell on the chronic cases. A case which has started acutely may become chronic if untreated, and although it may apparently recover spontaneously, it breaks out afresh from time to time, and this state of affairs may go on for years.

In older children one may get a condition more closely resembling the disease as manifested in adults. This is attended by a gradual onset passing on to anæmia, emaciation, and often extreme debility. Such cases suffer from periodical attacks of nausea and vomiting, with slight pyrexia, and may have enlargement of the liver and spleen. There are usually some urinary symptoms, and the urine is, of course, diagnostic. The prognosis of acute cases is eminently good, provided they are recognised early and properly treated. Relief of symptoms may be expected in the course of a very few days, and a cure established within a short time.

In cases which are or have become chronic, however, prognosis is less favourable, and they often require prolonged treatment before they are cured, while some cases succumb to the toxæmia in spite of all. In considering treatment, it must be remembered that relief of symptoms does not constitute a cure; the latter can only be assumed when the urine is clear of pus and bacilli.

In 1902 John Thomson published twenty-five cases which were treated by alkalies (potassium citrate and sodium bicarbonate), and in all cases the symptoms were relieved. He supposed that the alkalinity of the urine induced by these drugs was inimical to the growth of the Bacillus Coli, and that a cure was thus accomplished. Since then, however, it has been found that this "alkaline treatment" relieves the symptoms, but by no means always frees the urine from pus and bacilli, and Jordan has conclusively shown that the *Bacillus Coli* can grow as well in an alkaline as in an acid medium, hence it is to be presumed that the "alkaline treatment" neutralises the toxins produced, which in some cases is sufficient to enable the patient to cope with the infection and throw off the disease. Of late years Thompson Walker has shown that coliuria may be cured, at any rate in acute cases, by the administration of hexa-methylene-tetramine, combined with agents which will render the urine acid, and that this only fails if the urine is insufficiently acid, and that then the best agent for obtaining acidity of the urine is acid sodium phosphate, which may be pushed to large doses without ill-effects.

Thus, given a case with severe symptoms, the urine should be rendered alkaline until such symptoms have subsided, and then hexa-methylene-tetramine and acid sodium phosphate given until the urine is free from pus and organisms, remembering that the latter are chemically incompatible, and therefore must not be dispensed in the same bottle.

While such treatment will cure most cases, some chronic cases will not yield to it, and in these vaccines provide a valuable adjuvant—drug treatment should not be discontinued—but vaccines must be autogenous, as only 30 per cent. of cases are due to the true *Bacillus Coli,* most of the other members of the group being the causal organism in certain cases. Further, large doses are required ; one may commence with a dose of ten millions, but it is rare that good results are obtained under fifty millions, and it is sometimes necessary to proceed to 200 or 400 millions before a cure can be effected.

